# Microendoscopic discectomy versus minimally invasive transforaminal lumbar interbody fusion for lumbar spinal stenosis without spondylolisthesis

**DOI:** 10.1097/MD.0000000000020743

**Published:** 2020-06-12

**Authors:** Weihong Yi, Yu Tang, Dazhi Yang, Wenhua Huang, Huan Liu, Ziqi Sun, Yuan Yao, Yue Zhou

**Affiliations:** a Department of Orthopedics, the 6^th^ Affiliated Hospital of Shenzhen University Health Science Center, Shenzhen, Guangdong; b Department of Orthopedics, Xinqiao Hospital, Army Military Medical University, Chongqing; c The Precision Medicine Institute, the Third Affiliated Hospital, Southern Medical University, Guangzhou, Guangdong; d Jiebao Biotechnology Corporation; e Department of Orthopedics, Wuhan Fourth Hospital; Puai Hospital, Tongji Medical College, Huazhong University of Science and Technology, Wuhan, Hubei, China.

**Keywords:** decompression, fusion, lumbar spine, micoendoscopic discectomy (MED), minimally invasive spinal surgical methods, minimally invasive transforaminal lumbar interbody fusion (MIS-TLIF), spondylolisthesis, stenosis

## Abstract

Micoendoscopic discectomy (MED) and minimally invasive transforaminal lumbar interbody fusion (MIS-TLIF) has become alternatives of the traditional open decompression surgery alone and decompression plus fusion surgery in the treatment of lumbar spinal stenosis (LSS). To date, there is no study focusing on the comparison of clinical outcomes after MED and MIS-TLIF for LSS without spondylolisthesis.

Four hundred ninety-seven patients who underwent MED (236 cases) or MIS-TLIF (261 cases) for LSS without spondylolisthesis were included in this study. Perioperative outcomes (hospital stay, operation time and blood loss), cost, functional scores (Oswestry Disability Index, 12-item short form health survey) with a 24-month follow-up visit, complication and reoperation condition within 24 months after surgery were recorded and assessed.

No significant difference of clinical outcomes over time was observed between these 2 surgical approaches. Compared with MIS-TLIF, MED was associated with greater satisfaction at 1-month time point postoperatively, whereas this effect was equalized at 3-month time point postoperatively. MED brought advantages in shorter hospital stay, shorter operation time, less blood loss, and less cost over MIS-TLIF.

There was no significant difference in 24-month function scores over time between MED group and MIS-TLIF group. Compared with MIS-TLIF, MED could result in a better perioperative effect and less cost.

## 1 Introduction

Lumbar spinal stenosis (LSS) is a degenerative spondylotic disease caused by a gradual narrowing of spinal canal, resulting in compression on nerve root or cauda equina. Intermittent claudication is the typical LSS symptoms, which severely restrict quality of life. In cases of LSS, surgical treatment can obtain more satisfactory clinical outcomes than conservative treatment.^[1–3]^ The main surgical methods for LSS are decompression surgery alone and decompression plus fusion surgery.^[4]^

For LSS with spondylolisthesis, the selection of surgical methods is controversial. Some researchers considered decompression plus fusion surgery as a better selection than decompression surgery alone, with the intention of reducing the potential risk of spinal instability and deformity in the future and improving overall physical health-related quality of life.^[5–7]^ And some researchers reported that decompression plus fusion surgery did not lead to better clinical outcomes than decompression surgery alone.^[8,9]^ For LSS without spondylolisthesis, however, the evidence exhibiting the advantage of decompression plus fusion surgery or decompression surgery alone is still lacking. Recently, minimally invasive spinal surgical methods have been developed to improve the preservation of normal surrounding anatomical structures.^[10]^ Microendoscopic discectomy (MED, a bilateral decompression via a unilateral approach following conventional bilateral dissection of the paraspinal muscles and wide laminectomy) and minimally invasive transforaminal lumbar interbody fusion (MIS-TLIF) have become alternatives of the traditional open decompression surgery alone and decompression plus fusion surgery in the treatment of LSS.^[11–15]^ To date, there is no study focusing on the comparison of clinical outcomes after MED and MIS-TLIF for LSS without spondylolisthesis. In this study, functional scores over time (within 24 months postoperatively), perioperative outcomes, cost, complication and reoperation rate of patients after MED and MIS-TLIF for LSS without spondylolisthesis were compared, seeking to explore some helpful insights into preoperative decision.

## 2 Methods and materials

### 2.1 Ethical statement

This study was approved by the Medical Ethics Committee of Xinqiao Hospital, Amy Military Medical University, which was performed in accordance with the ethical standards of the 1964 Declaration of Helsinki. Written informed consent was obtained from every participant.

### 2.2 Patients

From January 2011 to May 2017, 497 patients who suffered LSS without spondylolisthesis underwent minimally invasive spinal surgery (MED, 236 cases; MIS-TLIF, 261 cases) in Department of Orthopedics, Xinqiao Hospital, Amy Military Medical University.

The inclusion criteria were as follows:

1. Intermittent claudication;
2. 1 or 2 adjacent lumbar stenotic segments (cross-section area of dural sac measuring 75 mm^2^ or less) on magnetic resonance imaging (MRI);
3. Duration of symptoms ≥ 3 months;
4. Conservative treatment was invalid.

The exclusion criteria were as follows:

1. Spondylolisthesis;
2. Instability of lumbar vertebrae;
3. Degenerative lumbar scoliosis (Cobb angle > 20 degrees);
4. History of lumbar spinal surgery;
5. Stenosis was not caused by degeneration;
6. Other specific spinal conditions, such as fracture, ankylosing spondylitis, and tumor in affected segments.

For all the included patients, each was considered to be appropriate for either MED or MIS-TLIF. Patients were fully informed of the details regarding these 2 minimally invasive spinal surgical methods, including the surgical procedures, advantages/disadvantages, risk of complication/recurrence, and cost. The final decision was dependent on the results of discussion between patients and surgeons.

### 2.3 Clinical assessment

Besides the demographic data (age, gender, body mass index [BMI], American Society of Anesthesiologist [ASA] score), each included patient was asked to completed a questionnaire including Oswestry Disability Index (ODI) and 12-item short form health survey (SF12, consisting of physical component summary [PCS] and mental component summary [MCS]) before surgery and at each time-point of follow-up visit. The follow-up visits were conducted at the time-point of 1, 3 , 6, 12, 18, and 24 months postoperatively. The patients received and returned the questionnaire via e-mail, mail or telephone.

The perioperative outcomes (operation time, blood loss, and hospital stay), total cost, complication, and reoperation condition were also collected to evaluate the clinical outcomes of the 2 minimally invasive spinal surgical procedures

The facet joint orientation of all the patients received MED was calculated according to the methods reported in previous study.^[16]^

In this study, there was a researcher group especially responsible for the collection and evaluation of the follow-up data. These researchers and the surgeons who performed surgery were blinded to the purpose of the study to reduce potential bias.

### 2.4 Surgical procedures and postoperative course

#### 2.4.1 MED

MED was performed with the METRx system. Several tubal dilators were inserted through an approximately 16 mm-long skin incision which was made to target the corresponding interlaminar space. The endoscope-assisted technology allows bilateral decompression through unilateral approach to decompress the bilateral recesses and central canal. Decompression was performed with long curved high-speed drills or Kerrison rongeurs. Decompression of the bilateral recesses was performed using medial facetectomy. The integrity of facet joint was then preserved with curved Kerrison rongeurs.

#### 2.4.2 MIS-TLIF

A paravertebral incision was made approximately 5 cm lateral to the midline of body:

1. Several dilators were inserted sequentially through the incision towards the facet complex to obtain a desired working diameter;
2. Facetectomy was carried out with high-speed drills to expose the desired posterolateral section of the disc;
3. The tubular retractor could be adjusted for decompression of stenosis;
4. Laminotomy and space distraction;
5. Bone graft was then inserted to the interbody space;
6. Contralateral decompression;
7. Percutaneous instrumentation of the pedicle screws was performed with fluoroscopy which could ensure the satisfactory positioning.

Off-bed activities were permitted to be performed under the protection of waist support after the removal of the drainage tube (2–5 days postoperatively). The patients could return to non-manual work 2 weeks after surgery. And they were allowed to perform full activity 3 months postoperatively. Heavy manual work was prohibited.

#### 2.4.3 Statistical analysis

The data was presented as the mean ± standard deviations (SD) or median (range). The changes in the parameters (ODI, SF12-PCS/MCS) over time between the 2 groups and within the same group were identified using repeated-measures analysis of variance. Independent-sample *t* test was employed to compare variables between 2 groups in cases with normal distributions. Mann–Whitney *U* test were employed to identify differences between 2 groups in cases with non-normal distributions. Chi-squared test were used to confirm differences of categorical variables between 2 independent groups. Significance value was set at a *P*-value of < .05. All data analyses were carried out with the software SPSS 20.0 (SPSS, Chicago, IL).

## 3 Results

### 3.1 Preoperative baseline characteristics

We reviewed 236 patients in MED group and 261 patients in MIS-TLIF group who met the inclusion criteria. The preoperative baseline characteristics were not significantly different between the 2 groups (Table 1).

**Table 1:**
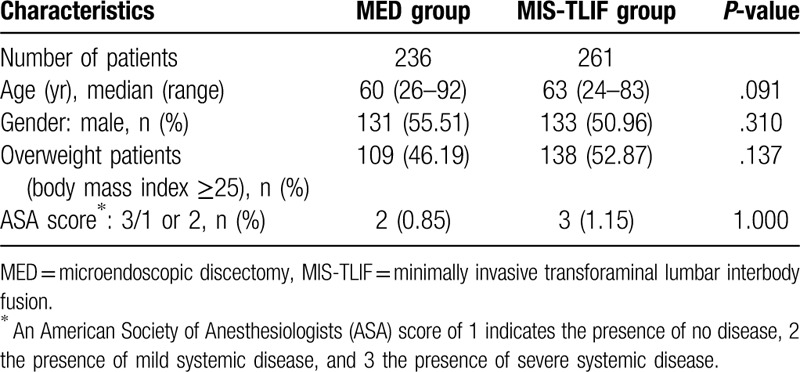
Baseline characteristics of the included patients.

### 3.2 Clinical outcomes

**Figure 1:**
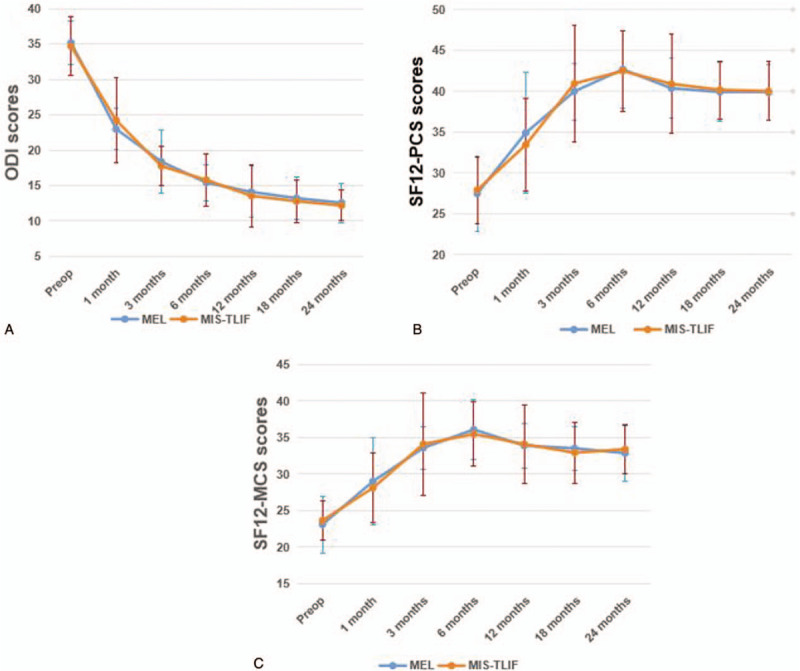
Functional scores of microendoscopic discectomy and minimally invasive transforaminal lumbar interbody fusion at different time points. MED = microendoscopic discectomy, MIS-TLIF = minimally invasive transforaminal lumbar interbody fusion.

Preoperatively, the ODI scores were 35.19 ± 3.13 in MED group and 34.69 ± 4.15 in MIS-TLIF group respectively. These results exhibited no significant difference (*P* = .129). After the surgery, the ODI scores reduced significantly in both the 2 groups compared with those in preoperative phase. The ODI scores in MED group were lower than those in MIS-TLIF group at 1-month time point (*P* = .004) after surgery. However, generally, there was no significant difference between the 2 groups in ODI scores over time (*P* = .364) (Table 2, Fig. 1A).

**Table 2:**
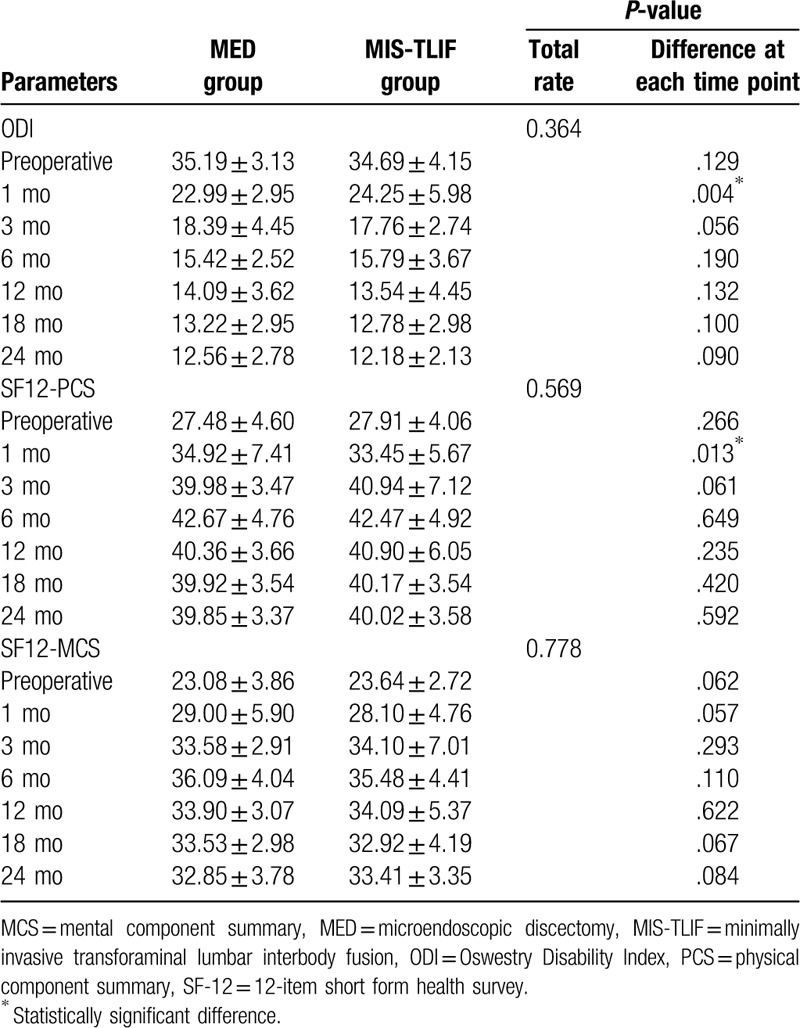
Clinical outcomes according to the parameters.

The preoperative SF12-PCS scores and SF12-MCS scores were 27.48 ± 4.60 and 23.08 ± 3.86 in MED group, 27.91 ± 4.06 and 23.64 ± 2.72 in MIS-TLIF group. These results showed no significant difference (*P* = .266 for PCS and *P* = .062 for MCS). After the surgery, both SF12-PCS scores and SF12-MCS scores increased significantly compared with those in preoperative phase. The SF12-PCS scores in MED group were higher than those in MIS-TLIF group at 1 month time point (*P* = .013) postoperatively. However, generally, there was no significant difference between the 2 groups for both SF12-PCS scores (*P* = .569) and SF12-MCS scores (*P* = .778) over time (Table 2, Fig. 1B and C).

### 3.3 Perioperative outcomes and cost

The mean operation time in MED group (76.31 ± 35.02 minutes) was significantly shorter than that in MIS-TLIF group (164.93 ± 59.59 minutes) (*P* < .001). There was no measurable intraoperative blood loss in MED group, and the blood loss was 181.53 ± 152.62 mL in MIS-TLIF group. The hospital stay in MED group (12.66 ± 6.33 days) was significantly shorter than that in MIS-TLIF group (14.35 ± 6.39 days) (*P* = .003). The total cost for in MED group (17050.39 ± 6281.01 Chinese Yuan) was significantly less than that in MIS-TLIF group (76315.05 ± 13265.06 Chinese Yuan) (*P* < .001). (Table 3)

**Table 3:**
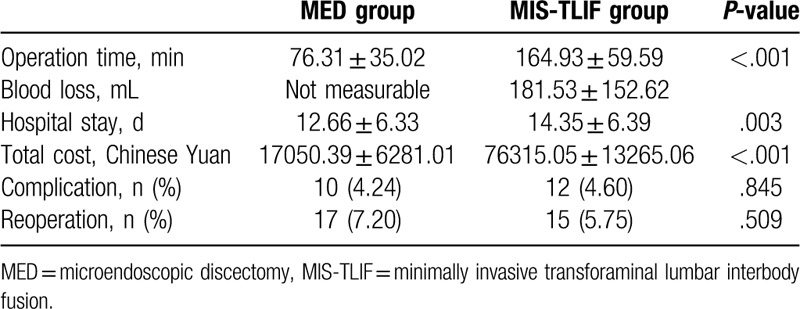
Summary of perioperative outcomes, cost, complications, and reoperation rate.

### 3.4 Complication and reoperation

Complications occurred in 10 patients (4.24%) in MED group and 12 patients (4.60%) in MIS-TLIF group. Eight patients in MED group and 12 patients in MIS-TLIF group suffered cerebrospinal fluid (CSF) leakage caused by incidental durotomy which had been restored intraoperatively and did not develop any symptoms needing further operation. In addition, 2 patients in MED experienced nerve root injury which caused transient unilateral cauda equina syndrome, no further treatment was performed, and the symptom disappeared after 1 to 3 weeks with sufficient rest. No major complication, such as retroperitoneal hematoma, wound infection and severe neurovascular injury, was observed. No significant difference in complication rate between these 2 groups was observed (*P* = .845). (Table 3)

Seventeen patients in MED group (7.20%) received reoperation, all of which were due to the subsequent spinal instability of the previously operative level. 15 patients in MIS-TLIF group (5.75%) received reoperation, all of which were due to adjacent lumbar disease. No significant difference in reoperation rate between these two groups was observed (*P* = .509). (Table 3)

Among the 17 patients who received reoperation after MED due to the subsequent spinal instability, 11 patients suffered lateral recess stenosis and 6 patients suffered central stenosis. There was no foraminal stenosis in this study. The left and right facet joint orientations of these 17 patient was listed in Table 4. Among the 236 patients who received MED, there was no significant difference of facet joint orientation between patients who received reoperation and patients who did not received reoperation (left: *P* = .143; right: *P* = .080).

**Table 4:**
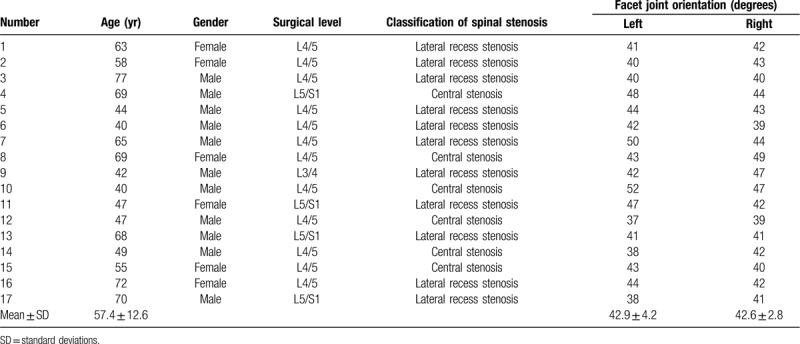
Additional medical information of 17 patients who received reoperation after microendoscopic discectomy due to the subsequent spinal instability.

## 4 Discussion

Traditionally, the main surgical methods for LSS without spondylolisthesis are decompression surgery and decompression plus fusion surgery. With the development of endoscope-assisted techniques, minimally invasive spinal surgical technology has been developed to reduce the damage to surrounding tissues, MED and MIS-TLIF have become alternatives of open decompression surgery and decompression plus fusion surgery.^[11–15]^ In this study, the comparison of clinical outcomes after MED and MIS-TLIF for LSS without spondylolisthesis was conducted, seeking to explore some helpful insights into the preoperative selection for the treatment of LSS.

Generally, in this study, all the functional scores used for the assessment of clinical outcomes were significantly improved postoperatively compared with those in preoperative stage in either MED group or MIS-TLIF group. The clinical status improved dramatically at 1 and 3 months postoperatively, reached and stayed at a relatively stable level at the period of 6 to 24 months. ODI score is responsive to the clinical status of the patients. A reduction of 15 points of ODI score could reflect obvious clinical improvement, which was proposed by the Food and Drug Administration.^[17]^ SF12-PCS/MCS scores were used to evaluate the health-related quality of life.^[18]^ In this study, there was no significant difference between the 2 groups for both ODI and SF12-PCS/MCS scores over time. Noticeably, the symptom-relief effect of patients in MED group was better than those in MIS-TLIF group at 1 month postoperatively, then the clinical status tended to reach the same level thereafter. This phenomenon may result from the relatively larger injury of soft tissues and disruption of spinal stability caused by interbody fusion and instrumentation than decompression surgery alone.

Consistent with the previous studies,^[19]^ the hospital stay and operation time of patients in MED group were significantly shorter than those in MIS-TLIF group, and the intraoperative blood loss and the cost of the MED surgery were less than those of the MIS-TLIF surgery. Additionally, compared with the data about traditional open decompression surgery and decompression plus fusion surgery,^[8]^ these 2 minimally invasive spinal surgery methods (MED and MIS-TLIF) showed better perioperative outcomes (shorter hospital stay, shorter operation time, and less blood loss), and less cost, which may be instrumental for both the physiological and psychological recovery after surgery. Thus, we believe that minimally invasive spinal surgery is a good choice for the treatment of LSS.

In this study, 8 patients in MED group and 12 patients in MIS-TLIF group suffered CSF leakage caused by incidental durotomy. Incidental durotomy is a common complication of spinal surgery.^[20]^ The occurrence of this complication is highly involved with the inherent difficulty in the use of tubular retractors. The limited visual field and narrow space can influence the maneuverability of the relevant instruments. These complications may be closely associated with the surgeons’ proficiency of the endoscope-assisted technique. The learning curves of either MED or MIS-TLIF is very deep. Expert knowledge of spine anatomy and abundant surgical experience are necessary to ensure safety. Therefore, we think that sufficient training including weekly case-discussion conferences, practical case teaching and live operation demonstration should be provided, especially for novice surgeons. In addition, 2 patients in MED experienced nerve root injury which caused transient unilateral cauda equina syndrome. This complication may result from the insufficient laminectomy, the insufficient decompression space may lead to transient compression on cauda equine.

In Ghogawala's study, the reoperation rate of open decompression surgery and decompression plus fusion surgery for LSS with spondylolisthesis was 34% and 14%, respectively, and there was significant difference of reoperation rate between the 2 groups.^[6]^ In Peter's study, the reoperation rate of open decompression surgery for LSS without spondylolisthesis was 21%.^[8]^ In Minamide's study, the reoperation rate of MED for LSS without spondylolisthesis was 9.9%.^[11]^ In this study, however, the reoperation rate after MED and MIS-TLIF for LSS without spondylolisthesis was much lower (7.20% and 5.75% respectively), and no significant difference between these 2 groups was observed. We think the reasons resulting in the difference of these studies may be as follows:

(1) The inherent spinal stability of patients with spondylolisthesis is worse than those without spondylolisthesis, the potential factors may still exist even after surgery;
(2) Compared with open spinal surgical methods, minimally invasive spinal techniques have advantages of preserving spinal stability;
(3) The follow-up period of this study was relatively short (24 months), some reoperation cases may occur in the next few years. We regard this point as a limitation of this study.

In clinical practice, when we decide if we select decompression alone or spinal fusion for the patient with LSS, one of the considerations is whether we can adequately decompress the spinal stenosis without affecting the stability of the spine. Some surgeons will decide according to:

(1) the morphology of the facet joint of the index level and
(2) if the stenosis extended to the foraminal level.

Blumenthal et al showed that facet joint angles were thought to be radiographic predictors for secondary instability and reoperation after decompression without fusion for low-grade spondylolisthesis.^[21]^ Grobler et al showed that case-specific assessment of residual facet joint morphology after decompression in both spinal stenosis and degenerative spondylolisthesis patients should be integrated into decisions about fusion for stability at the L4 to L5 level.^[16]^ However, Schär RT reported that facet joint orientation showed no significant difference between patients with and without reoperation after decompression of LSS.^[22]^ Thus, the impact of facet joint morphology on spinal instability is controversial. In the process of clinical decision, we will pay close attention to the facet joint morphology, but we do not regard relatively large facet joint angle in coronal dimension as contraindication for MED. We have reviewed the CT results of the 236 patients who received MED. We observed that there was no significant difference on the facet joint angle in coronal dimension between the patients who received reoperation and those who did not received reoperation. We think that this phenomenon could be attributed to the following reasons:

(1) all the cases in this study were diagnosed as lumbar spinal stenosis without spondylolisthesis or instability of lumbar vertebrae, which may lead to infrequent occurrence of the predictors for secondary instability;
(2) the sample size was too small (only 17 cases), thus it was insufficient to perform meaningful analysis.

In clinical practice, we usually classified LSS according to location (i.e., central, lateral recess and foraminal). Among the 17 patients who received reoperation after MED due to the subsequent spinal instability, 11 patients suffered lateral recess stenosis and 6 patients suffered central stenosis. There was no foraminal stenosis in this study. Although foraminal stenosis was not contraindication for MED, we tended to perform MIS-TLIF when treating the patients of this type, which will lead to the increase of bias. To avoid bias, all the patients included in this study were appropriate for either MED or MIS-TLIF theoretically. We think further comparative study in which LSS cases were segregated into 1 group with lateral recess stenosis only and the other group with lateral recess and foraminal stenosis is needed, which will be helpful to preoperative decision.

The sample size of this study is relatively small (MED 236 cases and MIS-TLIF 261 cases), which should be considered as limitation of this work. Based on these 497 cases, we have tried our best to avoid bias. We compared the baseline characteristics of these 2 groups. Then we chose appropriate statistical methods to identify differences between these 2 groups. Therefore, we believed that the lack of significant difference was not related to insufficient number of cases.

## 5 Conclusion

This study compared the outcomes of MED and MIS-TLIF for the patients who suffered LSS without spondylolisthesis. Compared with MIS-TLIF, MED was associated with greater satisfaction at 1-month time point postoperatively, whereas this effect was equalized at 3-month time point postoperatively, and there was no significant difference of the clinical outcome over time between these 2 minimally invasive surgical methods. Both of these 2 surgical methods provided ideal clinical effect, MED brought advantages in shorter hospital stay, shorter operation time, less intraoperative blood loss, and less cost over MIS-TLIF.

## Acknowledgments

We thank Professor Yanqi Zhang from Department of Statistics for statistical consultation.

## Author contributions

WY and YT: conceived the study, collected the data and participated in the analysis of samples; DY, WH and HL: performed the statistical analysis and participated in the design of the study; ZS: drafted the manuscript and performed the statistical analysis; YY and YZ: conceived and designed the study, collected the data and drafted the manuscript.
